# Coherent diffractive imaging of proteins and viral capsids: simulating MS SPIDOC

**DOI:** 10.1007/s00216-023-04658-y

**Published:** 2023-04-04

**Authors:** Thomas Kierspel, Alan Kadek, Perdita Barran, Bruno Bellina, Adi Bijedic, Maxim N. Brodmerkel, Jan Commandeur, Carl Caleman, Tomislav Damjanović, Ibrahim Dawod, Emiliano De Santis, Alexandros Lekkas, Kristina Lorenzen, Luis López Morillo, Thomas Mandl, Erik G. Marklund, Dimitris Papanastasiou, Lennart A. I. Ramakers, Lutz Schweikhard, Florian Simke, Anna Sinelnikova, Athanasios Smyrnakis, Nicusor Timneanu, Charlotte Uetrecht, for the MS SPIDOC Consortium

**Affiliations:** 1 Centre for Structural Systems Biology (CSSB), Deutsches Elektronen-Synchrotron DESY, Notkestraße 85, 22607 Hamburg, Germany; 2 Leibniz Institute of Virology (LIV), Martinistraße 52, 20251 Hamburg, Germany; 3 Institute of Microbiology of the Czech Academy of Sciences - BIOCEV, Průmyslová 595, Vestec, 252 50 Czech Republic; 4 European XFEL, Holzkoppel 4, 22869 Schenefeld, Germany; 5 Manchester Institute of Biotechnology and Department of Chemistry, The University of Manchester, Manchester, M1 7DN UK; 6 Department of Physics and Astronomy, Uppsala University, Box 516, 75120 Uppsala, Sweden; 7 Department of Chemistry – BMC, Uppsala University, Box 576, 75123 Uppsala, Sweden; 8 MS Vision, Televisieweg 40, 1322 AM Almere, Netherlands; 9 Centre for Free-Electron Laser Science, Deutsches Elektronen-Synchrotron DESY, Notkestraße 85, E22607 Hamburg, Germany; 10 Faculty V: School of Life Sciences, University of Siegen, Adolf-Reichwein-Str. 2a, 57076 Siegen, Germany; 11 Fasmatech, Technological and Scientific Park of Attica Lefkippos, NCSR DEMOKRITOS Patr, Gregoriou E’ 27, Neapoleos Str. 153 41, Agia Paraskevi, Attica, Greece; 12 University of Applied Sciences Technikum Wien, Höchstädtpl. 6, 1200 Vienna, Austria; 13 Institut Für Physik, Universität Greifswald, Felix-Hausdorff-Str. 6, 17489 Greifswald, Germany; 14 https://www.ms-spidoc.eu

**Keywords:** SPI, X-ray, Native MS, Protein complex structure, Viral particles, Simulation, Modeling

## Abstract

MS SPIDOC is a novel sample delivery system designed for single (isolated) particle imaging at X-ray Free-Electron Lasers that is adaptable towards most large-scale facility beamlines. Biological samples can range from small proteins to MDa particles. Following nano-electrospray ionization, ionic samples can be *m/z*-filtered and structurally separated before being oriented at the interaction zone. Here, we present the simulation package developed alongside this prototype. The first part describes how the front-to-end ion trajectory simulations have been conducted. Highlighted is a quadrant lens; a simple but efficient device that steers the ion beam within the vicinity of the strong DC orientation field in the interaction zone to ensure spatial overlap with the X-rays. The second part focuses on protein orientation and discusses its potential with respect to diffractive imaging methods. Last, coherent diffractive imaging of prototypical *T* = 1 and *T* = 3 norovirus capsids is shown. We use realistic experimental parameters from the SPB/SFX instrument at the European XFEL to demonstrate that low-resolution diffractive imaging data (*q* < 0.3 nm^−1^) can be collected with only a few X-ray pulses. Such low-resolution data are sufficient to distinguish between both symmetries of the capsids, allowing to probe low abundant species in a beam if MS SPIDOC is used as sample delivery.

## Introduction

Unraveling the structure–function relationship of biomolecules is of fundamental scientific interest. Due to its complexity, complementary approaches such as X-ray or electron diffraction, nuclear magnetic resonance (NMR) spectroscopy, or cryogenic electron microscopy are applied to measure the structure of, for example, proteins and non-covalently bound protein complexes such as viral capsids. Importantly, structural data is mostly available from a single condition creating a training bias for structure prediction, resulting in an insufficient understanding of underlying structural dynamics and hence limited clues on structural ensembles and transient states present in the solution.

Nowadays, mass spectrometry (MS) covers much more than simply determining the mass of a protein to very high accuracy [1]. Techniques like collision-induced dissociation (CID) and MS/MS approaches can disentangle the primary structure of proteins; ion mobility (IM) or hydrogen–deuterium exchange (HDX) MS allows access to the tertiary structure of proteins. In native MS, the structure of the protein complex is preserved, making it a useful tool to dissect quaternary structures. Therefore, MS provides a dynamic view on structures at multiple levels. To achieve atomic resolution imaging, the above-mentioned imaging (X-ray, electron) and spectroscopic (NMR) techniques are used nowadays. While these techniques are all very powerful and useful, each of them has its own strengths and weaknesses ranging from limitations of molecular size, through predominantly static snapshots of dynamic structures to bias towards highly abundant and energetically more favorable species.

In a novel approach, we aim to use MS techniques as sample delivery for imaging techniques like coherent diffractive imaging (CDI) to combine the advantages of both approaches. The development of this technique is the core of the Horizon 2020–funded research project MS SPIDOC (mass spectrometry for single-particle imaging of dipole-oriented complexes) [2].

Single-particle imaging (SPI) is an experimental technique at X-ray free-electron lasers (XFELs) where multiple (up to millions) isolated particles are imaged individually, and their diffraction patterns are added to retrieve a full 3D diffraction image [3–5]. The approach hence resembles SPI in cryo-EM. Nowadays, SPI experiments at XFELs primarily utilize aerodynamic-focusing injectors as sample delivery systems [3]. MS SPIDOC provides three major benefits. It allows *m/z*-selectivity to ensure that the recorded diffraction patterns belong to the same species. The orientation of proteins can reduce the number of required diffraction patterns [6], and no gas is required to focus and decharge the sample, which significantly reduces the number of background photons. In addition, nano-ESI allows for very low sample consumption (nL/min), which is especially important for precious biological samples. MS SPIDOC is designed for the SPB/SFX instrument at the European XFEL [7]. However, the parts specific to the implementation of this instrument are not shown here, and its modularity allows for easy adaption to other beamlines and facilities.

Here, we show a front-to-end simulation of the entire setup for a possible diffraction experiment. In “Results: ion trajectory simulations,” we present the setup of MS SPIDOC and use this as a basis to discuss the performed ion trajectory simulations. A closer and more detailed look of the ion trajectory simulations can be found in the deliverable of the MS SPIDOC project [8]. “Results: protein orientation” describes how proteins are oriented in external direct current (DC) fields. Finally, simulated SPI of biologically relevant norovirus capsids is shown and discussed in “Results: diffraction.” It shows that MS SPIDOC is a versatile sample delivery system for selective probing of transient or low abundant species with X-ray pulses, be it diffraction or fragmentation as readout.

## Results: ion trajectory simulations

**Fig. 1:**
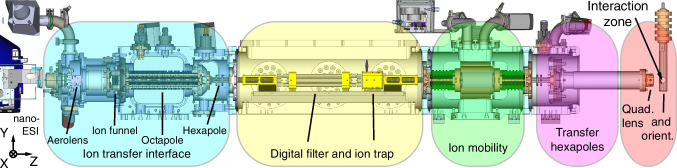
CAD model of the MS SPIDOC setup. From left to right: ions are transferred into the gas phase by using nano-ESI and introduced in the system by the ITI interface. The ions can be filtered, trapped, and structurally separated via ion mobility before they are sent to the interaction zone using the transfer hexapoles. A quadrant lens is used as a last ion optical element to steer the ions before they enter the dipole orientation lens through a hole (Fig. 2). The interaction zone is in the middle of the dipole orientation electrode

Figure 1 shows a computer-aided design (CAD) of the MS SPIDOC setup. Highlighted are the individual components, namely the ion transfer interface (ITI), the digital ion filter and trap (DIT), the ion mobility chamber (IM), the transfer hexapole, the quadrant lens, and the dipole orientation electrode (DO). In addition, a nano-ESI source in the form of the Triversa Nanomate (Advion) is shown. The Triversa Nanomate is a robotized sample delivery system, which can also be replaced by a static nano-ESI. The displayed setup is the standard configuration of MS SPIDOC and used for the front-to-end simulation. Based on specific applications, the modularity of the setup allows for the interchanging or removal of individual components.

Ion trajectory simulations have been conducted for the different segments individually via Simion [9] or SIMAX [10]. Ion trajectory handover between the modules has typically happened at the intersections between the modules by providing the phase space distribution of the individual ion ensembles. However, handover has also been tested at other positions within each module with no significant influence on the outcome of the trajectory simulations.

The simulations were performed on different cluster sizes (*n*) and charge states (*m*) of cesium iodide [(CsI)_n_Cs]^m+^ (3, 3.3, and 6.1 kDa), ubiquitin (8.6 kDa, Ubi^1+^ and Ubi^6+^), hemoglobin (64 kDa, Hb^13+^), and chaperonin GroEL (808 kDa, GroEL^62+^). The samples were represented by hard spheres and their corresponding *m/z* values.

The first module that has been modeled was the ITI, which is composed of the aerolens™ [11], the ion funnel, a combined octapolar/quadrupolar ion guide, and a hexapole. The aerolens is designed to increase the transmission efficiency during the transport of ions into the gas phase. Due to the high-pressure region within the aerolens, gas-flow dynamics were used for the simulations. Starting from the ion funnel (ITI), the ions were treated with an initial axial velocity of 500 m/s. The gas pressure (*N*_2_) was set to 2 mbar in the funnel area, and between 10^−3^ and 10^−2^ mbar in the octapole region to improve the thermalization of the ions by collisions with the background gas. Thermalization of the ion motion is important to reduce ion losses as non-thermalized ions primarily exhibit losses through transversal movement [12]. The simulations showed that especially heavier species like GroEL^62+^ profit from elevated pressures and an additional trapping time of up to 5 ms within the octapole region. After thermalization, the ions are transferred with a near-zero eV ion beam through the second aperture following the ITI octapole into the ITI hexapole and guided towards the DIT module. The near-zero kinetic energy enables thereby again an efficient transmission and trapping of the ions in the DIT module.

The DIT consists of a quadrupole filter, an ion trap, and the input and output transfer hexapoles. A detailed description of this module can be found in [13]. In short, the DIT is driven by a digital radiofrequency (RF) signal, i.e., the electric field is switched between ground and a positive voltage at a frequency of several hundred kHz. The duty cycle can be chosen freely and is 50% in normal transmission mode to mimic the fundamental of a sinusoidal frequency. The digital guiding field has two main advantages: (i) it allows for fast tuning over a wide *m/z* range as it does not rely on resonant circuits; (ii) the broad mass range used for this setup would have required at a certain point altered hardware with sinusoidally driven fields. In general, the DIT can be used in transmission as well as trapping mode. In both cases, the digital filter can be set to a broad (several thousand) *m/z* range, or to individual or multiple adjacent peaks of the spectrum. Peak selection is achieved by tuning the frequency and the duty cycle of the driving RF field. Typically, mass-resolving power *R*^1^ comes at the cost of transmission, ranging from close to 100% transmission for *R* < 100 down to transmission of 15% for *R* ≈ 700. Filtering besides selectivity reduces the risk of space charge effects in the trap. Within the trap region, buffer gas can be applied for thermalization purposes and hence efficient trapping over prolonged times of 100 ms. This trapping duration is important since the initial operating mode of MS SPIDOC is designed based on the X-ray pulse structure of the European XFEL, i.e., a dark time of 99.4 ms, followed by a 0.6 ms burst of X-rays. In addition, a pulsed ionic beam is also necessary for spatial separation in the ion mobility device.

The ion mobility module was specifically designed to separate different conformers of large biomolecules and their complexes. This was achieved by modifying an ion mobility system from [14], i.e., a modified, high-fidelity resistive glass drift tube. The simulations were conducted with a few mbar of pressure (*N*_2_) inside the drift cell and showed that a transmission of above 95% is achieved for species larger than 1450 m*/z*, for which the device is primarily designed*.* At 556 m*/z* (leucine enkephalin), the transmission drops to 20%. A resolution of 20 can realistically be achieved for the arrival time distributions, although this is partially masked by broad conformational ensembles observed in proteins.

**Fig. 2:**
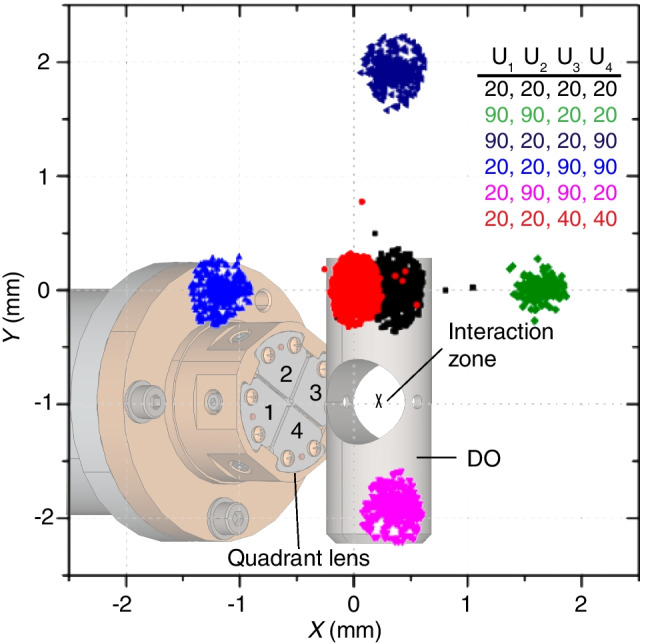
Simulated ion beam profiles of GroEL^62+^ in the interaction zone for different voltage (V) configurations of the quadrant lens (U_1_–U_4_) showing the deflection in mm relative to the center of the DO. For all configurations, the DO electrode voltage was set to −50 kV. A CAD inset highlights the four segments of the quadrant lens, including the DO

Subsequently, the ions are transported to the X-ray interaction zone via a transfer hexapole and a quadrant lens. The latter can be used to steer the ion beam transversely and overlap it with intersecting X-rays in the center of the DO (Fig. 2). This steering capability is essential for fine alignment, since it is much easier to translate the sample position compared to moving the X-rays. In the given setup with −50 kV high acceleration voltage for dipole orientation, the ion position can be shifted by several mm, i.e., several times the ion beam diameter, by applying comparably low voltages between 20 and 90 V.

## Results: protein orientation

**Fig. 3:**
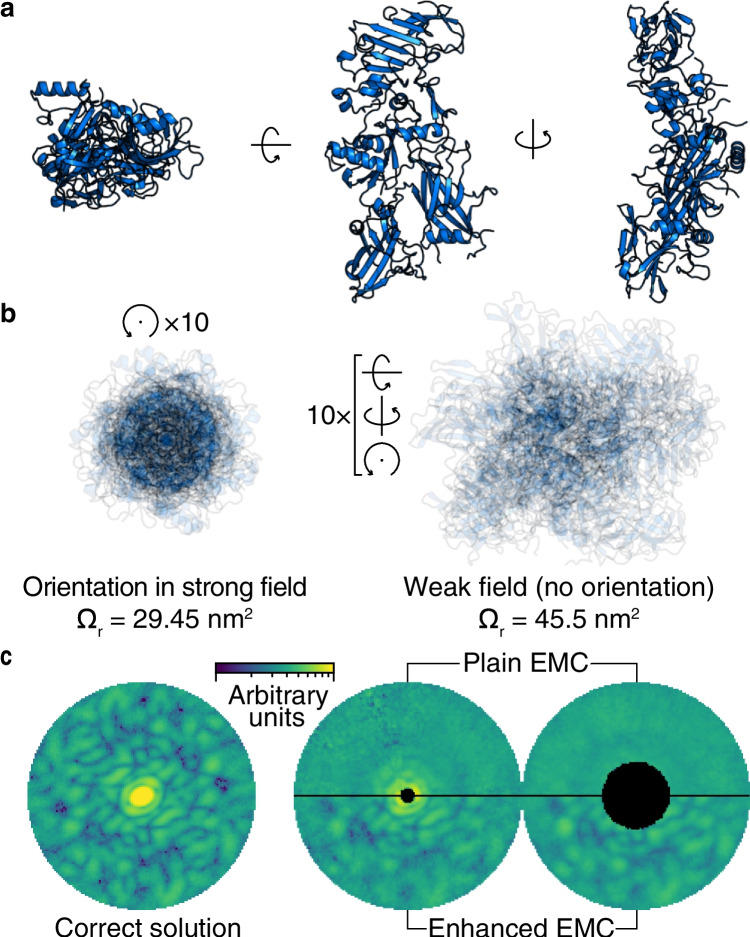
Exemplifying the benefit of Enhance EMC. **a** Three perpendicular views of an asymmetric protein, the anthrax protective antigen (PDB code 1ACC). **b** Overlays of 10 random rotations around the longest axis, and 10 random 3D rotations, illustrate the oriented and non-oriented case, respectively. The substantial differences between directional cross section, *Ω*_d_, and rotationally averaged cross section *Ω*_r_ (calculated from thousands of rotations) imply the large differences that for example SAXS experiments would sample. **c** Comparison of the performance between standard EMC and EMC enhanced with dipole orientation. The left pattern shows the correct solution. The middle pattern is a slice through the output from EMC using a low number of diffraction patterns (3000), showing that only the enhanced version converges in this case. The right plot is a similar comparison with a larger number of patterns (10,000) but where the beam stop was made too large for plain EMC (figure adopted from [6])

The orientation of proteins as discussed in the following utilizes the fact that most proteins do have an intrinsic permanent dipole moment. By applying an external DC electric field, the protein can orient such that its dipole moment orients along the external field lines. Such an orientation can be beneficial for X-ray imaging techniques such as SPI and conventional X-ray scattering methods like small-angle X-ray scattering (SAXS). Considering SPI, controlling the orientation in one dimension, the problem of combining the diffraction patterns during 3D electron density reconstruction could be substantially reduced [6]. Adding the knowledge about orientation to the so-called Expand, Maximize, and Compress (EMC) computer algorithms [15] used for clustering, the diffraction patterns could have three advantages: (i) speeding up the algorithms by faster convergence; (ii) reducing the number of required diffraction patterns, thus saving sample and experimental time; and finally (iii) finding convergence in some case where it would be impossible without prior knowledge on orientation—for example, when there is missing data in the diffraction patterns due to the detector geometry. The first case is relevant for large and complex data sets, whereas (ii) and (iii) represent common situations where the number of useful hits is low or when the beam stop/gaps in the detector array precludes data collection in certain areas. This method of taking advantage of a known angle is generally called enhanced-EMC, or EEMC (Fig. 3).

In SAXS, the orientation would potentially provide more structural information than current conventional methods. For a protein with a non-spherical form, it is in principle possible to get information on the extension of the molecules along more than one direction (Fig. 3).

**Fig. 4:**
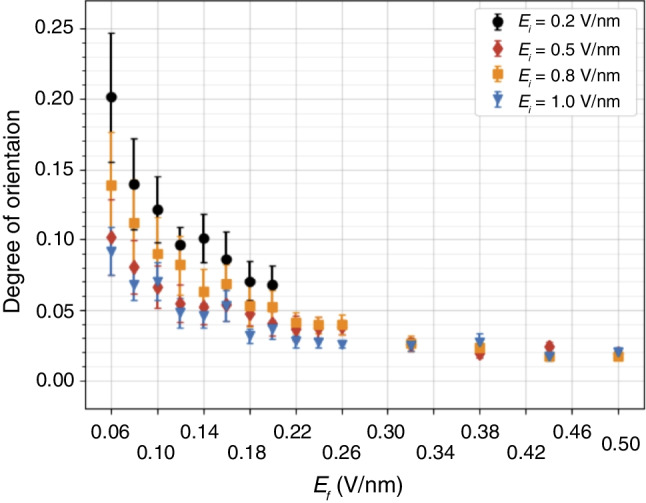
Degree of dipole orientation in combined electric fields. Average degree of dipole orientation over the last 0.5 ns for all combinations of initial electric field strengths *E*_*i*_, and final electric field strengths *E*_*f*_. A result of 0.00 degree indicates consistent and full orientation of the protein. Molecular dynamics simulations were done on ubiquitin in vacuum similarly as in [16]. The huge field strength was used to speed up simulations and is not applicable to experimental use as discussed below

Molecular dynamics simulations predict that it is possible to take advantage of the intrinsic dipole moment present in most proteins to orient the protein using electric fields [6]. These simulations show that static field strength between 0.05 and 1.5 V/nm indeed orients four simulated proteins (ubiquitin, lysozyme, ctf, Trp-cage) without significantly distorting their structure. In a follow-up study, a more realistic simulation setup was used, where the electric field was not turned on immediately but rather gradually increased as the protein ion approaches the orientation device, or as the voltage switches from 0 to *V*_Max_. Slowly ramping up the field seems to better preserve the molecular structure of the protein [16], which is an advantage in any imaging experiment. In the case of fields that are strong enough to unfold the protein structure, the protein first orients and then unfolds, following the principle of “orientation before destruction.” This finding indicates the possibility to use at first a stronger field for orientation, followed by a weaker field to maintain the orientation. Using molecular dynamics simulations and following the simulation protocol described in [16], we have investigated this, and the results are presented in Fig. 4. The simulations were done with the ubiquitin protein and two different electric field strengths. First, a higher field *E*_*i*_ was applied for 1 ns, followed by a lower field *E*_*f*_ for 1 ns. The quality of the orientation is defined using the degree of orientation, *D* = 1 −  < cosθ > , where *θ* is the averaged angle between the field and the total dipole of the molecules. Both field strengths/durations are below the value where a significant structural change is expected [6], and more details on the simulations can be found in [16, 17]. The results show that it is to some extent possible to first orient the molecule in a stronger field and then keep the orientation using a weaker field. Therefore, it could be beneficial to only shortly expose the protein to strong fields for structural preservation. Alternatively, a well-timed X-ray pulse could capture the oriented structure before the destructive effect of the field can manifest.

## Results: diffraction

The MS SPIDOC setup is designed to deliver biological samples into the XFEL beam, to generate an interpretable X-ray diffraction image. While the ultimate goal is to be able to mass- and conformationally select transient species from solutions, to initially demonstrate the benefit of sorting particles based on size, we focused on two geometrical configurations of norovirus-like particles, the *T* = 1 (3 MDa) or *T* = 3 (10 MDa) capsids, which are accessible to native MS and can be separated by their *m/z* [18, 19]. We have used these known structures as a starting point to calculate the expected diffraction signal from a single-pulse XFEL exposure. Simulations were performed using the tool package SIMEX [20] developed at the European XFEL. We used experimental parameters relevant to the X-ray pulse and detector as listed in Table 1. All photons are assumed to interact with the sample at the same time, i.e., the X-ray pulse duration is zero and is providing therefore an instantaneous diffraction pattern.

**Table 1:** XFEL pulse and detector parameters corresponding to what is available at SPB/SFX

X-ray pulse	Detector
Photon energy: 6 keV	Distance to detector: 1 m
Pulse energy: 3 mJ	Pixel size: (400 μm)^2^
Focal spot diameter: 1 μm	No. of pixels: 512^2^

**Fig. 5:**
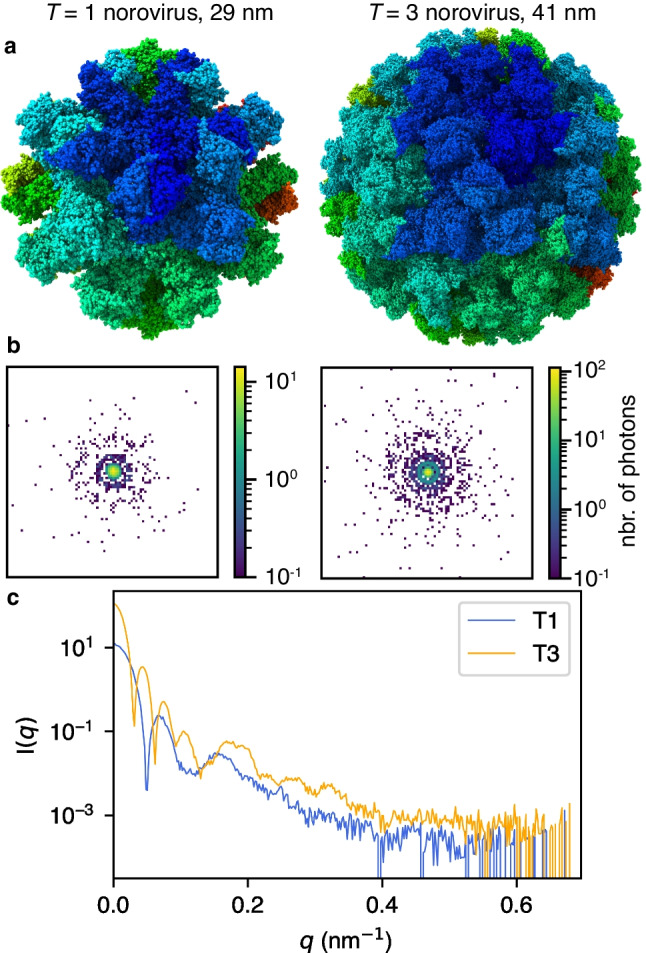
Simulated diffraction patterns of norovirus capsids.** a** Illustration of the 3D structure of the two simulated capsid samples of GII.2 Snow Mountain norovirus strain in the two geometries—*T* = 1 (pdb:6ouc, left) and *T* = 3 (pdb:6otf, right). **b** Examples of the calculated diffraction patterns with Poisson noise. **c** Average integrated detector signal vs *q* = 1/*d*, where *d* is the spatial resolution. Averages are over ten diffraction images from different exposure angles. To illustrate the effect of detector noise, Poisson noise has been applied to each of the diffraction images. The first minimum in the diffraction from the larger capsid *T* = 3 appears at around *q* = 0.03 nm^−1^, whereas it is 0.05 nm^−1^ for the smaller *T* = 1 capsid. Any noise due to sample heterogeneity or radiation that is on the length scale of 1 nm or below will not affect the ability to distinguish between the *T* = 1 and the *T* = 3 capsid

We simulated ten diffraction patterns from each of the two capsids, each time with the capsid in a new, random orientation. Examples of the diffraction patterns can be seen in Fig. 5. To estimate if it is possible to distinguish the two capsids apart based purely on diffraction data, we calculated the total diffracted signal in the ten individual patterns, and then integrated over 2π. This way we can compare the expected diffracted signal as a function of the scattering angle for the two systems. This would, in an experiment, allow for online sorting without the need for reconstruction, and it would allow us to study the particle sorting sensitivity in the MS SPIDOC instrument.

We expect that there are two major types of structural dynamics that will influence the diffracted signal: the sample heterogeneity, i.e., how different each of the *T* = 1 or *T* = 3 capsids from each other, and sample dynamics due to radiation damage. A recent study showed the impact that the structural fluctuations of two globular proteins would have on the achievable resolution in an XFEL SPI experiment using all-atom classical molecular dynamics simulations [21], as implemented in the GROMACS simulation package [22]. Lysozyme and ubiquitin were simulated in a vacuum, at temperatures ranging from 250 to 350 K. Diffraction signals were calculated using a package developed by [23], using the same principles as SIMEX. From the study done on the two globular proteins, it can be concluded that the expected resolution limit due to structural fluctuations is below 2 Å, at temperatures up to 350 K. This fluctuation is well below the resolution needed to see the differences between *T* = 1 and *T* = 3.

In *T* = 1 and *T* = 3, we have multiple copies of the same proteins forming together the large non-covalently bound capsid assembly, whereas in the aforementioned study of the two globular proteins, we investigated only separate monomeric proteins. We expect that the resolution limit of the two norovirus capsids is less affected by structural fluctuations than the two globular proteins, since a repetition of units both increases the stability of the system through increased contacts and the ways to dissipate excess energy, as well as increases the scattered X-ray signal.

Radiation damage is the other source of structural dynamics that could affect the ability to image the capsids. The effect of the ionization on the speckle contrast, directly linked to the resolution, was investigated by [24]. This study was done on a 14 kDa globular protein (lysozyme), for which we expect that the photo-induced Coulomb explosion is faster than for systems as large as capsids. It was concluded that speckle contrast from lysozyme is more sensitive to natural structural variations than to the radiation damage induced from pulses with a pulse duration of up to 50 fs; a relatively long duration pulse at most XFELs.

Based on our simulations, we can conclude that the initial imaging part of the proposed experiment is feasible. We should initially be able to detect the differences between the *T* = 1 and *T* = 3 capsids. The noise we expect from radiation damage and structural fluctuations has no significant influence on the resolution required to distinguish the two capsids. Moreover, large structural changes due to ionization and gas-phase transfer are unrealistic without significant activation [25]. The structural integrity has further been proven in complementary approaches using native MS for the preparation of *m/z* selected protein complexes for electron microscopy [26, 27]. Our conclusions are in line with earlier bio-imaging experiments at the European XFEL [5, 28], which support the notion that we should experimentally be able to detect at least the two first scattering rings from the two capsids.

As the research field is developing very fast, it is difficult to estimate the achievable resolution in a future experiment. This is mainly due to the background and achievable number of snapshot frames currently being the largest limiting factor in such experiments [3]. Optimizations of both factors are being investigated by multiple groups, and the progress is very rapid. MS SPIDOC contributes by providing a background-free sample delivery system with the capability for future advanced experimental schemes.

## Conclusion and outlook

The ion trajectory simulations define the setup parameters of the prototype and provide useful information with regard to good experimental parameters for the individual components. A simple quadrant lens can be used to overlap the ion beam with the X-rays even in the presence of a strong DC orientation field. Moreover, protein orientation (or in general sample orientation) can be very beneficial for CDI experiments like SPI or SAXS. DC orientation fields have a minimal influence on the protein structure. In addition, the orientation can likely be maintained in weaker electric fields after orientation in initial stronger DC electric fields. This combination allows further reduction of any adverse influence on protein structure. SPI simulations with realistic XFEL parameters show that *T* = 1 and *T* = 3 norovirus capsids provide enough scattering signal to distinguish between both symmetries on the basis of just a handful diffraction patterns, and are therefore also very promising candidates for first proof-of-principle experiments.

In general, MS SPIDOC provides unique features as a sample delivery system:

1. The ITI is used to significantly increase the ion flux for kDa-MDa protein samples, allowing further reduction of the estimated measurement times for SPI from [29].
2. Due to the charge of ions, *m/z* filters can be applied to select relevant species from more complex mixtures, which is a key feature considering the innate heterogeneity of many biological samples. In addition, ion mobility can be used to further reduce the structural heterogeneity of the sample by conformational or topological separation.
3. The MS-based sample delivery systems allow providing of samples in the absence of background scatterers such as gases or liquids.
4. The possibility to orient proteins in the interaction zone increases the information content that can be gained by CDI experiments.

MS SPIDOC is a sample delivery system specifically designed for the measurement of biologically relevant samples at X-ray facilities. In contrast to much simpler aerodynamic-focusing-based sample delivery systems *used for SPI at XFELS*, MS SPIDOC utilizes state-of-the-art MS-based techniques to pre-select (size and conformation) and orient the sample. These measures promise a significant reduction of measurement time, opening thereby the possibility to study a much broader range of biological sample*s*,* including low abundant species*,* transient states*, *and mixtures of samples.* Apart from static structure measurements, MS SPIDOC is already set up to measure viral particles and their intermediate species as exemplified in other native MS studies [30]. Last but not least, the sample delivery system can also be used for sample delivery beyond CDI. Of specific interest is native top-down MS where the X-rays can be used to fragment protein complexes and thereby circumvent intrinsic problems of bottom-up MS caused by post-digestion analysis of the proteins’ primary structure.

## Footnotes

1 *R* = (*m/z*)/(Δ*m*/*z*), where Δ*m/z* is the minimum peak separation which allows to distinguish two ion species.
